# Effects of different light intensity on leaf color changes in a Chinese cabbage yellow cotyledon mutant

**DOI:** 10.3389/fpls.2024.1371451

**Published:** 2024-04-16

**Authors:** Jianyu Huo, Ninan Zhang, Ying Gong, Yongrong Bao, Yinyin Li, Lugang Zhang, Shanshan Nie

**Affiliations:** State Key Laboratory of Crop Stress Resistance and High-Efficiency Production, College of Horticulture, Northwest A&F University, Yangling, Shaanxi, China

**Keywords:** Chinese cabbage, yellow cotyledon mutant, leaf color, light intensity, carotenoid, RNA-seq

## Abstract

Leaf color is one of the most important phenotypic features in horticultural crops and directly related to the contents of photosynthetic pigments. Most leaf color mutants are determined by the altered chlorophyll or carotenoid, which can be affected by light quality and intensity. Our previous study obtained a Chinese cabbage yellow cotyledon mutant that exhibited obvious yellow phenotypes in the cotyledons and the new leaves. However, the underlying mechanisms in the formation of yellow cotyledons and leaves remain unclear. In this study, the Chinese cabbage yellow cotyledon mutant 19YC-2 exhibited obvious difference in leaf color and abnormal chloroplast ultrastructure compared to the normal green cotyledon line 19GC-2. Remarkably, low-intensity light treatment caused turn-green leaves and a significant decrease in carotenoid content in 19YC-2. RNA-seq analysis revealed that the pathways of photosynthesis antenna proteins and carotenoid biosynthesis were significantly enriched during the process of leaf color changes, and many differentially expressed genes related to the two pathways were identified to respond to different light intensities. Remarkably, *BrPDS* and *BrLCYE* genes related to carotenoid biosynthesis showed significantly higher expression in 19YC-2 than that in 19GC-2, which was positively related to the higher carotenoid content in 19YC-2. In addition, several differentially expressed transcription factors were also identified and highly correlated to the changes in carotenoid content, suggesting that they may participate in the regulatory pathway of carotenoid biosynthesis. These findings provide insights into the molecular mechanisms of leaf color changes in yellow cotyledon mutant 19YC-2 of Chinese cabbage.

## 1 Introduction

Leaf is an important source of plant photosynthesis which is critical for plant growth and development (Brodribb et al., 2007). Various leaf color is significant for enhancing the ornamental effect of plant species and is an important quality trait for fruits and vegetables (Zhao et al., 2020). Leaf color is mainly determined by the types and contents of pigments, such as chlorophyll and carotenoid, which are two major photosynthetic pigments (Markwell and Namuth, 2003). Chlorophyll is the most important pigment in photosynthesis and responsible for capturing light energy and energy conversion (Fromme et al., 2003; Eckhardt et al., 2004). Carotenoid is natural light capture pigment and transfers absorbed light to chlorophyll, and it also plays vital roles in photoprotection against oxidative damage (Frank and Cogdell, 1996; Hashimoto et al., 2016). Leaf color mutants are widespread in plants, and one of the widely available mutation types is leaf yellowing. Numerous yellow leaf mutants have been identified and reported in many vegetable crops, such as pepper (Yang et al., 2023), watermelon (Xu et al., 2023), cucumber (Zhang et al., 2022a), wucai (Nie et al., 2021), and Chinese cabbage (Zhang et al., 2017; Zhao et al., 2021). Studies have demonstrated that yellow leaf phenotype of mutant is usually caused by the disrupted biosynthesis and degradation of chlorophyll and carotenoid (Zhao et al., 2020), and thus it is commonly used for studying the photosynthetic system, pigment biosynthetic pathway, and genetic regulatory mechanism.

The inhibition of chlorophyll photosynthesis or chlorophyll deficiencies caused leaf color changes involve a series of genes that regulate the complex chlorophyll biosynthetic pathway (Zhao et al., 2020). The first committed step of chlorophyll synthesis is mediated by magnesium chelatase (MgCh), which is a key enzyme in chlorophyll biosynthesis and catalyzes the insertion of Mg^2+^ into protoporphyrin IX (Masuda, 2008). Plant MgCh consists of three subunits, MgCh I (CHLI), MgCh D (CHLD), and MgCh H (CHLH), whose mutations have been extensively characterized in many plant mutants (Masuda, 2008). A yellow-green leaf mutant of strawberry (*Fragaria pentaphylla*) was reported to be attributed to the gene mutation of *CHLI*, which resulted in lower chlorophyll level in mutant (Ma et al., 2023). The mutation of *CHLH* in zucchini (*Cucurbita pepo*) caused the decrease in chlorophyll content and exhibited yellow peel phenotype (Niu et al., 2023). Virus-induced gene silencing of *CHLI* and *CHLD* in pea (*Pisum sativum*) led to the decrease in chlorophyll content and abnormal chloroplast structure and showed yellow leaf phenotype (Luo et al., 2013). With the roles of chlorophyll in leaf color changes being well studied, the effects of carotenoid biosynthesis on the genetic diversity of plant colors also receive the increasing research attention. Carotenoid biosynthetic pathway is highly regulated by the coordinated expression of many carotenogenic genes, which mediate the accumulation and degradation of different components of carotenoids (Cazzonelli and Pogson, 2010; Yuan et al., 2015). Studies in red or yellow tomato fruits and citrus showed that the increases in phytoene synthase gene (*PSY*) and phytoene desaturase gene (*PDS*) expressions contributed to the elevated carotenoid content (Kato et al., 2004; Cao et al., 2019; Chen et al., 2023a). The decreased expressions of downstream lycopene *β*-carotene (*LCYB*) and lycopene *ϵ*-cyclase (*LCYE*) genes in strawberry were reported to be related to the changes in *β*-carotene and lutein (Zhu et al., 2015). Moreover, a yellow-fleshed fruit tomato mutant was a consequence of the deletion of *PSY1* gene region and showed a reduction in carotenoid accumulation (Gady et al., 2012). In addition, previous studies had revealed that orange head Chinese cabbage accumulated significant higher amounts of carotenoids in leaves in comparison with white head Chinese cabbage (Watanabe et al., 2011; Zhang et al., 2015). Although many studies have explored the roles of carotenoid in determining fruit colors and leaf coloration, whether carotenoid metabolism is associated with yellow leaf mutation in leafy vegetables requires in-depth research.

Carotenoid metabolism is affected by various environmental stimuli (Yuan et al., 2015; Sun et al., 2018). Different light quality has been documented to affect the content and compound of carotenoid in many studies (Stanley and Yuan, 2019). For instance, blue light treatment can promote the synthesis and accumulation of lutein in strawberry and citrus (Chen et al., 2023b; Ma et al., 2021). Moreover, different light intensity or the presence of light has a significant impact on carotenoid production (Grumbach and Lichtenthaler, 1982; Stanley and Yuan, 2019). In bagged fruits of apple, light exclusion reduced the content of carotenoid, but it was alleviated by *PSY* overexpression in fruits (Ampomah-Dwamena et al., 2022). In green leaves of chili pepper, the absence of light was related to the low expressions of *PSY* and *PDS* genes and led to the low carotenoid content (Simkin et al., 2003). In white mustard (*Sinapis alba*), the upregulated expression of *PSY* gene resulted in an increase in carotenoid content under light conditions (Lintig et al., 1997). Notably, in the yellowing leaf mutant of pepper, high-intensity light was required to regulate the transition of leaves from green to yellow, which was positively related to the increased carotenoid pigments (Liu et al., 2023). Therefore, to investigate the light intensity induced changes in carotenoid content is necessary for understanding the underlying mechanisms of leaf color changes in yellow leaf mutants.

In this study, a yellow cotyledon mutant of Chinese cabbage (*Brassica rapa* L. ssp. *Pekinensis*) was used, and it was obtained through a natural mutation in the hybrid progenies of Chinese cabbage. To study the effects of different light intensity on leaf color changes in Chinese cabbage yellow cotyledon mutant, physiological characteristics and changes in chlorophyll and carotenoid contents were analyzed both in the cotyledons and leaves of mutant in the current study. Furthermore, RNA-seq analysis was performed to identify the critical differentially expressed genes (DEGs) involved in the leaf color changes in response to different light intensity. These results facilitate uncovering the regulatory mechanisms of leaf color changes in Chinese cabbage yellow cotyledon mutant.

## 2 Materials and methods

### 2.1 Plant materials and different light intensity treatments

Two genetically stable pure lines of Chinese cabbage yellow cotyledon mutant 19YC-2 and normal green cotyledon near isogenic line (wild type) 19GC-2 were used and provided by Cruciferae Research Group of Northwest A&F University (Yangling, China). The seedlings were grown in Caoxinzhuang vegetable experimental farm (Yangling, China). Five individual seedlings were selected for phenotype statistics at the cotyledon stage, the two-leaf stage, the four-leaf stage, the six-leaf stage, the rosette stage, and the heading stage, respectively. The shoots were harvested to measure the fresh weight at different stages and then dried at 65°C with 12 h for measuring the dry weight. Three independent replicates were performed for each sample at every stage.

For different light intensity treatments, the germinated seeds of 19YC-2 and 19GC-2 were sowed in plant growth chamber with 16 h light (24°C, 216 μmol·m^−2^·s^−1^), 8 h dark (16°C), and 55% relative humidity. After the cotyledon of seedlings were fully expanded, the seedlings were separately subjected to the conditions under low-intensity light (72 μmol·m^−2^·s^−1^) and normal intensity light (216 μmol·m^−2^·s^−1^) conditions, with the same other conditions. The leaf color observation and sampling were performed at the cotyledon stage, the first-leaf stage, the two-leaf stage, the three-leaf stage, and the four-leaf stage, respectively. The cotyledons and leaves for RNA extraction were collected, immediately frozen in liquid nitrogen and stored at −80°C.

### 2.2 Ultrastructural observation of chloroplast

Transmission electron microscopy (TEM) was performed to observe the chloroplast ultrastructure of the cotyledons and leaves of 19YC-2 and 19GC-2. The cotyledons were used from 19YC-2 and 19GC-2 at the cotyledon stage. The 1^st^ leaves of 19YC-2 and 19GC-2 were separately collected at the two-leaf stage and the four-leaf stage. The samples in size with 1 mm^2^ were fixed in a mixture of 2% glutaraldehyde fixative in 0.2 M phosphate buffered saline (PBS). The fixed samples were then dehydrated and embedded in resin as previously decreased (Nie et al., 2023). Ultrathin sections were obtained and examined using a transmission electron microscopy (Hitachi HT7800, Japan).

### 2.3 Measurement of color difference parameters

The color difference parameters of cotyledons and leaves were measured by a colorimeter (Konica Minolta CR-400, Japan). A CIE Lab color system was used to examine the L*, A* and B* values. The L* value represents leaf brightness. The A* value represents the red and green, where a positive value indicates red and a negative value indicates green. The B* value represents the yellow and blue, where a positive value indicates yellow and a negative value indicates blue.

### 2.4 Measurement of photosynthetic pigment and chlorophyll fluorescence

The contents of photosynthetic pigments were determined as previously reported methods (Lichtenthaler, 1987; Yang et al., 2023). The fresh leaves with 0.3 g were cut and mixed in a 50 mL tube containing 10 mL 95% ethanol in the dark for 24 h. The absorbance was measured by a M200pro microplate reader (Tecan, Switzerland) at wavelengths of 665 nm, 649 nm, and 470 nm, respectively. The contents of carotenoid, chlorophyll a, and chlorophyll b were calculated according to the reported formula by Yang et al. (2023). A Dual-PAM-100 Chlorophyll Fluorometer (Walz, Germany) was used to monitor the chlorophyll fluorescence parameters, including maximum quantum efficiency of photosystem II (Fv/Fm), effective quantum efficiency of photosystem II (Fv’/Fm’), and electron transport in photosystem II [ETR(II)] and nonphotochemical quenching (NPQ). Three individual seedlings were used in each replicate, and three independent replicates were performed for each sample.

### 2.5 RNA-seq analysis

A total of 18 cDNA libraries were constructed using six samples from 19GC-2 and 19YC-2, including the cotyledons (CK0 and T0) at the cotyledon stage, and the 4^th^ leaves after 72 μmol·m^−2^·s^−1^ (CK1 and T1) and 216 μmol·m^−2^·s^−1^ (CK2 and T2) light treatments at the four-leaf stage, with three independent biological replicates. The library construction and sequencing for RNA-seq analysis were completed on Illumina sequencing platform by Genedenovo Biotechnology Co., Ltd (Guangzhou, China). Clean reads were mapped to the reference genome of Chinese cabbage V3.0 (http://brassicadb.cn). Pearson correlation coefficients (PCC) and principal component analysis (PCA) were used to evaluate the correlation between samples and repeatability. The abundance of gene expression was quantified as fragments per kilobase of transcript sequence per millions (FPKM) value. The differentially expressed genes (DEGs) were identified using the DEseq 2 R package with the absolute value of log2 fold change ≥ 1 and FDR < 0.05 (Love et al., 2014). TB tools software was used to draw heat maps and Venn diagram. Functional annotation of gene was performed according to Gene Ontology (GO) and Kyoto Encyclopedia of Genes and Genomes (KEGG) databases, and enrichment analysis was performed at *P*-value ≤ 0.05. The data of RNA-seq were deposited in NCBI Sequence Read Archive (SRA; http://www.ncbi.nlm.nih.gov/Traces/sra/) database under the accession number: BioProject PRJNA1005457.

### 2.6 Quantitative real-time PCR

Total RNA extraction, cDNA synthesis, and qRT-PCR analysis were performed as previously reported methods (Nie et al., 2019). The qRT-PCR analysis was conducted on an iCycler iQ5 Real-Time PCR Detection System (Bio-Rad, USA). Three biological replicates were performed for each sample. The specific primer sequences were designed using Beacon Designer 8.0 software (**Supplementary Table S1**). The *BrEF-1α* gene (*Bra031602*) was selected as the internal control. The relative expression level of gene was calculated using 2^−ΔΔCT^ method.

### 2.7 Weighted gene co-expression network analysis

To construct a co-expression network and identify the modules of highly correlated DEGs based on the gene expression profiles and pigment contents, weighted gene co-expression network analysis (WGCNA) was performed using the R package with the soft threshold of 8 and the minimum module size of 50 (Langfelder and Horvath, 2008). Correlation analysis between the identified modules and the contents of photosynthetic pigments were determined by Pearson correlation coefficients (PCC).

### 2.8 Statistical analysis

All the data were analyzed using SPSS Statistics 24.0. The expression data were presented as the means ± SEM. Independent samples t-test was used to determine statistically significant difference.

## 3 Results

### 3.1 Phenotype characteristic and chloroplast ultrastructure of Chinese cabbage yellow cotyledon mutant

**Figure 1:**
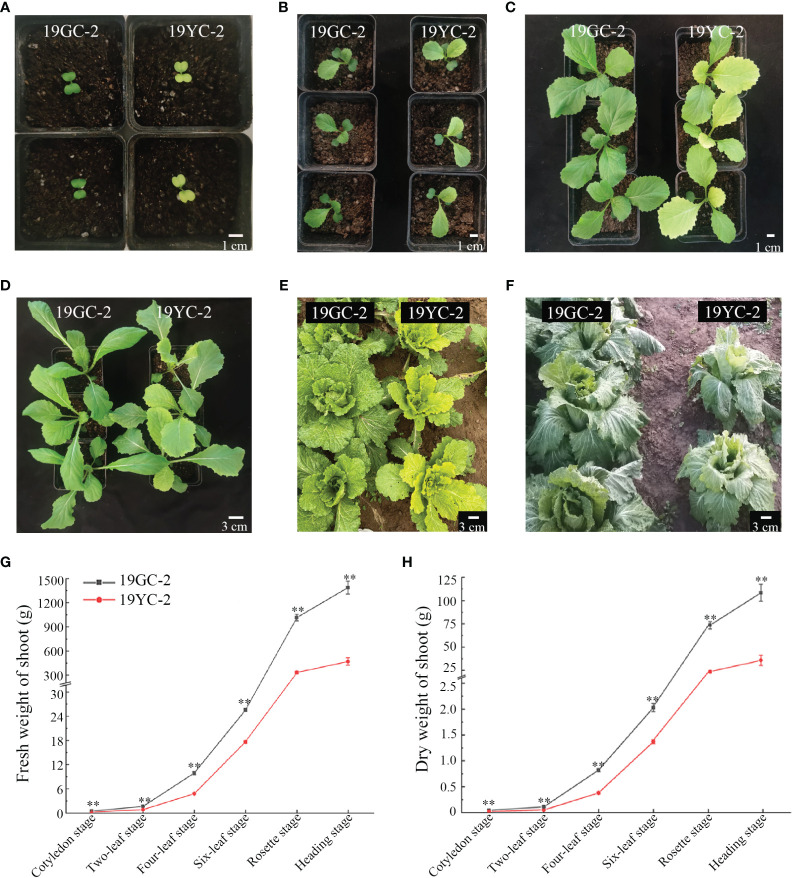
Leaf phenotypes in green cotyledon line 19GC-2 and yellow cotyledon mutant 19YC-2 of Chinese cabbage. **(A)** Cotyledon stage. **(B)** Two-leaf stage. **(C)** Four-leaf stage. **(D)** Six-leaf stage. **(E)** Rosette stage. **(F)** Heading stage. **(G)** Statistic of fresh weight of shoots in 19GC-2 and 19YC-2. **(H)** Statistic of dry weight of shoots in 19GC-2 and 19YC-2. Scale bar = 1 cm **(A–C)** and 3 cm **(D–F)**. **P < 0.01.

The leaf phenotypes of Chinese cabbage yellow cotyledon mutant 19YC-2 and green cotyledon line 19GC-2 were observed at different growth stages. The cotyledons and leaves of 19GC-2 were normal green from the cotyledon stage to the heading stage. The cotyledons of 19YC-2 were obvious yellow at the cotyledon stage (**Figure 1A**) and began to turn green at the two-leaf stage (**Figure 1B**). The 1^st^ leaves of 19YC-2 also exhibited yellow at the two-leaf stage (**Figure 1B**), while it gradually turned green at the four-leaf stage (**Figure 1C**). Interestingly, the newly unfolded leaves of 19YC-2 were still yellow from the four-leaf stage until the rosette stage (**Figures 1C-E**). At the heading stage, all the outer leaves of 19YC-2 were close to green (**Figure 1F**). In addition, the fresh weight and dry weight of shoots in 19YC-2 were significantly lower than that in 19GC-2 at each stage (**Figures 1G, H**).

**Figure 2:**
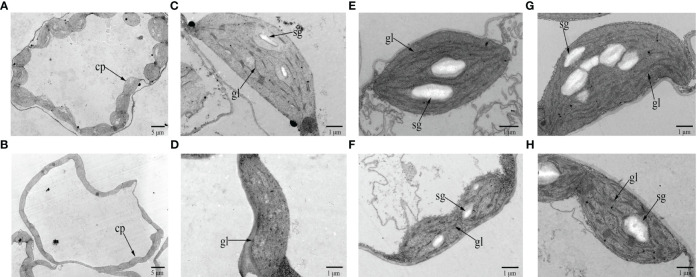
Chloroplast ultrastructure in the cotyledons and leaves of 19GC-2 **(A, C, E, G)** and 19YC-2 **(B, D, F, H)**. Observation of the number of chloroplasts per cell **(A, B)** and the chloroplast structure **(C, D)** at the cotyledon stage. The chloroplast structure in the 1^st^ leaves of 19GC-2 and 19YC-2 at the two-leaf stage **(E, F)** and at the four-leaf stage **(G, H)**. cp, chloroplast; gl, grana lamellae; sg, starch grain. Scale bar = 5 μm **(A, B)** and 1 μm **(C-H)**.

Observation of chloroplast ultrastructure by TEM showed that there were fewer chloroplasts per cell in the cotyledons of 19YC-2 (approximately 11 chloroplasts) than that in 19GC-2 (approximately 20 chloroplasts) (**Figures 2A, B**). The chloroplasts in 19GC-2 were regular and oval in shape, and the obvious grana lamellae and starch grains were observed (**Figure 2C**). However, the chloroplasts in 19YC-2 were irregular and shrunken in shape and exhibited the lack of grana lamellae and starch grains (**Figure 2D**). Moreover, at the two-leaf stage, the chloroplasts in the 1^st^ leaves of 19GC-2 were normal in shape and structure (**Figure 2E**), while the 1^st^ leaves of 19YC-2 were still yellow and exhibited abnormal chloroplasts and a lower number of grana lamellae and starch grains (**Figure 2F**). Remarkably, at four-leaf stage, the 1^st^ leaves of 19YC-2 had gradually turned green, and the chloroplasts exhibited the normal structures with obvious grana lamellae and increased starch grains (**Figure 2H**), which was almost similar to the normal chloroplasts in 19GC-2 (**Figure 2G**).

### 3.2 Leaf color changes of Chinese cabbage yellow cotyledon mutant under different light intensity

**Figure 3:**
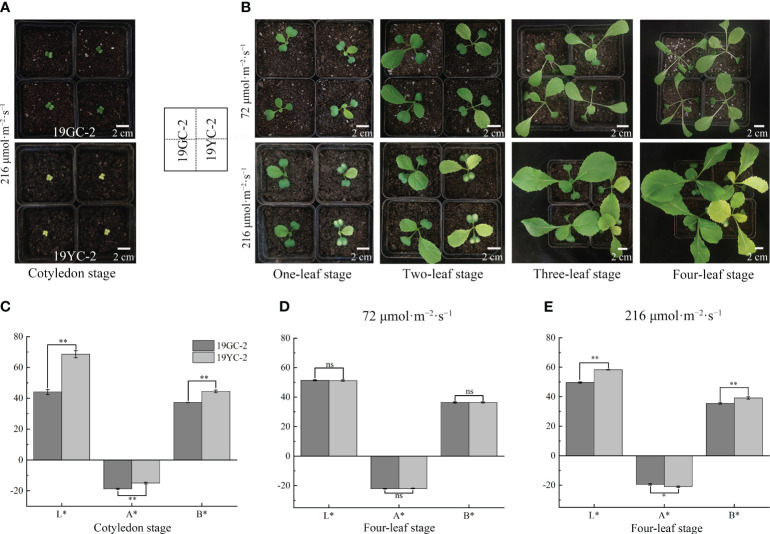
Effects of different light intensities on leaf colors in 19GC-2 and 19YC-2. **(A)** The cotyledons of 19GC-2 and 19YC-2 before treatment. **(B)** Leaf phenotypes of 19GC-2 and 19YC-2 at different growth stages after 72 μmol·m^-2^·s^-1^ and 216 μmol·m^-2^·s^-1^ light treatment. Color difference parameters of the cotyledons **(C)** at the cotyledon stage and the 4^th^ leaves at the four-leaf stage after 72 μmol·m^-2^·s^-1^ **(D)** and 216 μmol·m^-2^·s^-1^ **(E)** light treatments. The L*, A* and B* values represent the color difference parameters. The L* value represents leaf brightness. The A* value represents the red and green. The B* value represents the yellow and blue. Scale bar = 2 cm **(A, B)**. ns, no significant; *P < 0.05; **P < 0.01.

To explore the effects of different light intensities on leaf color changes in Chinese cabbage yellow cotyledon mutant 19YC-2, the two light intensities of 72 μmol·m^−2^·s^−1^ and 216 μmol·m^−2^·s^−1^ were separately used to treat the seedlings from fully expanded cotyledons (**Figure 3A**). Under 72 μmol·m^−2^·s^−1^ light condition, both 19YC-2 and 19GC-2 displayed thinner leaves. All the leaves of 19YC-2 gradually turned green until the four-leaf stage, which had few differences with the leaf color in 19GC-2 (**Figure 3B**). Under 216 μmol·m^−2^·s^−1^ light condition, the heart leaves of 19YC-2 remained yellow at the four-leaf stage (**Figure 3B**). Further analysis of color difference showed that the values of L *, A *, and B * in 19YC-2 cotyledons were significantly higher than that in 19GC-2 at the cotyledon stage (**Figure 3C**). After the treatment under 72 μmol·m^−2^·s^−1^ light, there was no significant difference in color difference parameters in the 4^th^ leaves between 19YC-2 and 19GC-2 at the four-leaf stage (**Figure 3D**). However, under 216 μmol·m^−2^·s^−1^ light condition, the significant differences in color difference parameters in the 4^th^ leaves between 19YC-2 and 19GC-2 were found at the four-leaf stage (**Figure 3E**). The results of color difference analyses were consistent with the observation of leaf color in 19YC-2 and 19GC-2 after different light intensity treatments.

### 3.3 Effects of different light intensity on pigment contents in Chinese cabbage yellow cotyledon mutant

**Figure 4:**
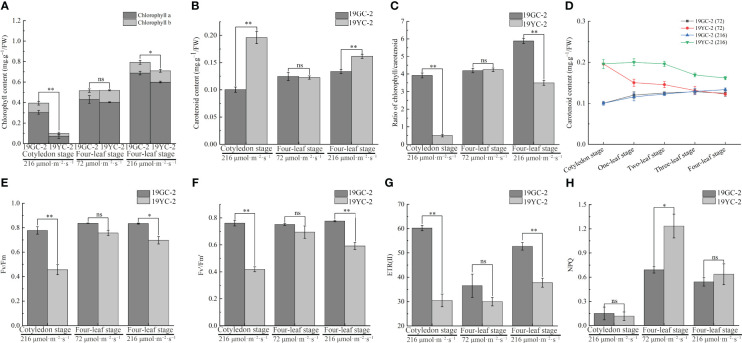
Effects of different light intensities on pigment contents and chlorophyll fluorescence parameters in 19GC-2 and 19YC-2. **(A)** Chlorophyll content. **(B)** Carotenoid content. **(C)** The ratio of chlorophyll/carotenoid content. **(D)** Changes in carotenoid content in the heart leaves of 19GC-2 and 19YC-2 at different stages. **(E)** Fv/Fm: The maximum quantum efficiency of photosystem II. **(F)** Fv’/Fm’: The effective quantum efficiency of photosystem II. **(G)** ETR(II): The electron transport in photosystem II. **(H)** NPQ: The non-photochemical quenching. ns, no significant; *P < 0.05; **P < 0.01.

Analyses of pigment contents showed that the chlorophyll content in 19YC-2 cotyledons was significantly lower than that in 19GC-2 (**Figure 4A**), and the carotenoid content in 19YC-2 cotyledons was significantly higher than that in 19GC-2 (**Figure 4B**). Furthermore, after the treatment using 72 μmol·m^−2^·s^−1^ light, the chlorophyll and carotenoid contents in the 4^th^ leaves of 19YC-2 had no significant difference compared to that in 19GC-2 at the four-leaf stage (**Figures 4A, B**). Under 216 μmol·m^−2^·s^−1^ light condition, the chlorophyll content was significantly lower, and the carotenoid content was significantly higher in the 4^th^ leaves of 19YC-2 than that in 19GC-2 at the four-leaf stage, which was consistent with the changes in pigment contents at the cotyledon stage (**Figures 4A, B**). Additionally, the ratio of chlorophyll/carotenoid in the 4^th^ leaves of 19YC-2 under 72 μmol·m^−2^·s^−1^ light was nearly equal to that in 19GC-2, while the significant lower ratio was found in the 4^th^ leaves of 19YC-2 under 216 μmol·m^−2^·s^−1^ light than that in 19GC-2 (**Figure 4C**), which may be responsible for the leaf color differences in 19YC-2 exposed to different light intensities. More importantly, the carotenoid content in the heart leaves of 19YC-2 was gradually decreased with the growth of seedlings (**Figure 4D**). The decrease trend of the level of carotenoid in 19YC-2 after 72 μmol·m^−2^·s^−1^ light treatment was more rapid, resulting in the nearly equal level of carotenoid with the 4^th^ leaves of 19YC-2 to that in 19GC-2. The results indicated that low-intensity light treatment caused the rapid decrease in carotenoid content in 19YC-2 may be one of the major reasons for the leaf color changes from yellow to green.

### 3.4 Effects of different light intensity on chlorophyll fluorescence parameters in Chinese cabbage yellow cotyledon mutant

Analyses of chlorophyll fluorescence parameters in Chinese cabbage yellow cotyledon mutant 19YC-2 showed that at the cotyledon stage, the values of Fv/Fm, Fv’/Fm’, and ETR (II) were significantly lower in 19YC-2 cotyledons than that in 19GC-2 (**Figure 4E-G**), while the NPQ had no difference between 19YC-2 and 19GC-2 (**Figure 4H**). The observation revealed that the yellow cotyledons in 19YC-2 exhibited obvious photoinhibition, which may be related to the abnormal chloroplasts and lower chlorophyll content in 19YC-2 cotyledons. After 72 μmol·m^−2^·s^−1^ light treatment, there was no significant difference in the values of Fv/Fm, Fv’/Fm’, and ETR (II) in the 4^th^ leaves between 19YC-2 and 19GC-2 at the four-leaf stage (**Figures 4E-G**), while the NPQ was significantly higher in 19YC-2 (**Figure 4H**), indicating that low-intensity light activated the NPQ and induced the raised energy dissipation to defense photodamage in 19YC-2. After 216 μmol·m^−2^·s^−1^ light treatment, the values of Fv/Fm, Fv’/Fm’, and ETR (II) were also significantly lower in the 4^th^ leaves of 19YC-2 at the four-leaf stage (**Figures 4E-G**), and the NPQ had no difference between 19YC-2 and 19GC-2 (**Figure 4H**), which was consistent with the results at the cotyledon stage.

### 3.5 RNA-seq analysis of Chinese cabbage yellow cotyledon mutant under different light intensity

**Figure 5:**
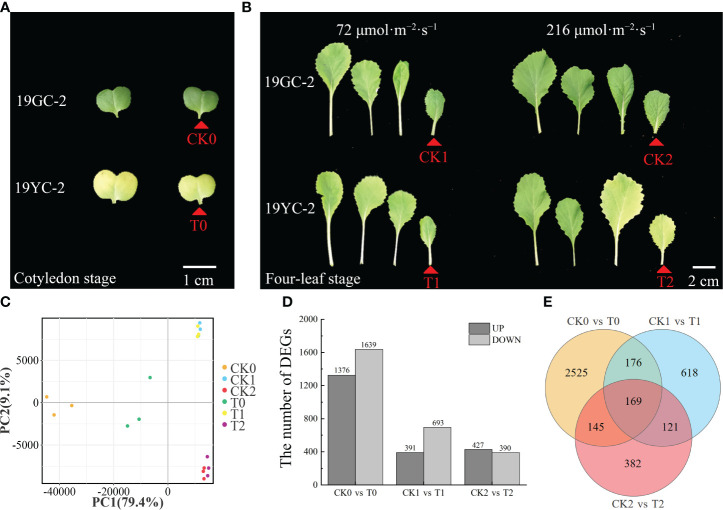
RNA-seq analysis of DEGs in 19GC-2 and 19YC-2. **(A)** The cotyledons of 19GC-2 and 19YC-2 at the cotyledon stage were used for constructing the cDNA libraries of CK0 and T0 (red arrowhead marked). **(B)** The leaves of 19GC-2 and 19YC-2 at the four-leaf stage after 72 and 216 μmol·m^-2^·s^-1^ light treatment. The 4^th^ leaves were used for constructing the cDNA libraries of CK1, CK2, T1, and T2 (red arrowhead marked). **(C)** Principal component analysis (PCA). **(D)** The number of DEGs. **(E)** Venn diagram of the unique and common DEGs among different libraries. Scale bar = 1 cm **(A)** and 2 cm **(B)**.

The cotyledons of 19GC-2 (CK0) and 19YC-2 (T0) and the 4^th^ leaves of 19GC-2 (CK1 and CK2) and 19YC-2 (T1 and T2) after 72 and 216 μmol·m^-2^·s^-1^ light treatment were used for constructing six cDNA libraries and RNA-seq analysis (**Figures 5A, B**). After filtering the original data, the average clean data ranged from 38.84 to 44.02 million were obtained in six cDNA libraries, the average GC contents were ranged from 47.17% to 48.46%, and the average Q30 values were ranged from 92.85% to 93.46% (**Supplementary Table S2**). These obtained reads were mapped to the reference genome of Chinese cabbage with the average comparison efficiency ranged from 91.75% to 92.78% (**Supplementary Table S3**). PCA analysis validated the high reliability among three biological replications in the six sample groups (**Figure 5C**). Statistics of the number of DEGs showed that 3015 DEGs were identified in the comparison group of CK0 vs T0 (1376 upregulated and 1639 downregulated DEGs), 1084 DEGs were identified in the comparison group of CK1 vs T1 (391 upregulated and 693 downregulated DEGs), and 817 DEGs were identified in the comparison group of CK2 vs T2 (427 upregulated and 390 downregulated DEGs) (**Figure 5D**). Additionally, 169 common DEGs were obtained among three comparison groups of CK0 vs T0, CK1 vs T1, and CK2 vs T2, 345 common DEGs were obtained among the comparison groups of CK0 vs T0 and CK1 vs T1, 314 common DEGs were obtained among the comparison groups of CK0 vs T0 and CK2 vs T2, and 290 common DEGs were obtained among the comparison groups of CK1 vs T1 and CK2 vs T2 (**Figure 5E**).

Functional annotation and enrichment analysis showed that these DEGs were classified into 48 (CK0 vs T0), 45 (CK1 vs T1), and 43 (CK2 vs T2) GO subcategories (**Supplementary Figure S1**). Moreover, 662 (CK0 vs T0), 224 (CK1 vs T1) and 171 (CK2 vs T2) DEGs were annotated on 119, 94, and 89 KEGG pathways (**Supplementary Figure S2**), respectively. Among these pathways, the metabolic pathway (ko01100) and biosynthesis of secondary metabolites (ko01110) showed significant enrichment in all the three comparison groups. Importantly, the pathways of photosynthesis antenna proteins (ko00196) and carotenoid biosynthesis (ko00906) showed significant enrichment in the comparison groups of CK0 vs T0 and CK2 vs T2, while the two pathways were not found in the comparison group of CK1 vs T1. In addition, DEGs related to porphyrin and chlorophyll metabolism (ko00860) was enriched only in the comparison group of CK0 vs T0, while no DEGs were found in CK1 vs T1 and CK2 vs T2. Furthermore, gene set enrichment analysis (GSEA) also showed that the pathways of photosynthesis antenna proteins (ko00196) and carotenoid biosynthesis (ko00906) exhibited striking enrichment in the comparison groups of CK0 vs T0 and CK2 vs T2, while not in CK1 vs T1 (**Supplementary Figure S3**). The results of enrichment analyses suggested that DEGs encoding photosynthesis antenna proteins and the differential expressed carotenoid biosynthesis genes may be directly related to the leaf color difference between 19YC-2 and 19GC-2 under different light intensity.

### 3.6 Identification of DEGs related to photosynthesis antenna proteins

**Figure 6:**
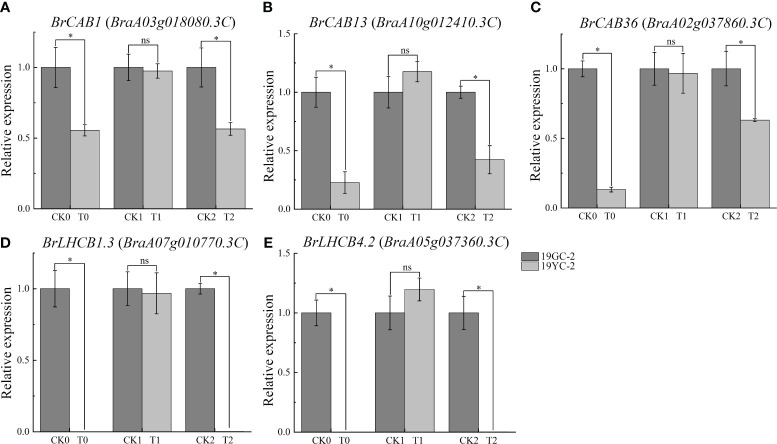
Expression analysis of DEGs related to photosynthetic antenna proteins by qRT-PCR in 19GC-2 and 19YC-2. **(A)** *BrCAB1* gene. **(B)** *BrCAB13* gene. **(C)** *BrCAB36* gene. **(D)** *BrLHCB1.3* gene. **(E)** *BrLHCB4.2* gene. CK0, T0, CK1, T1, CK2, and T2 represent the six cDNA libraries from 19GC-2 and 19YC-2. ns, no significant; *P < 0.05.

Photosynthetic antenna proteins had been documented to be capable of binding pigments, turning their excitation energies, and directing energy transfer to the reaction center of photosystem (Ruban, 2015). In this study, a total of 17 DEGs were identified and enriched in the pathway of photosynthesis antenna proteins (**Supplementary Table S4**). Expression analysis showed that 15 DEGs exhibited downregulated expression in the comparison group of CK0 vs T0, and 8 DEGs exhibited downregulated expression in the comparison group of CK2 vs T2, while no DEGs were identified in the comparison group of CK1 vs T1. Remarkably, the expression levels of six transcripts, including *BrCAB1* (*BraA03g018080.3C*), *BrCAB13* (*BraA10g012410.3C*), *BrCAB36* (*BraA02g037860.3C*), *BrLHCB1.3* (*BraA07g010770.3C*, *BraA07g010780.3C*), and *BrLHCB4.2* (*BraA05g037360.3C*), were significantly downregulated in both CK0 vs T0 and CK2 vs T2. To validate the differential expression of these DEGs, 5 genes were selected for qRT-PCR analysis. The results showed that all the selected genes were significantly decreased in T0 compared with CK0 and in T2 compared with CK2 (**Figure 6**), which was consistent with the results from RNA-seq analysis.

### 3.7 Identification of DEGs involved in carotenoid biosynthesis pathway

**Figure 7:**
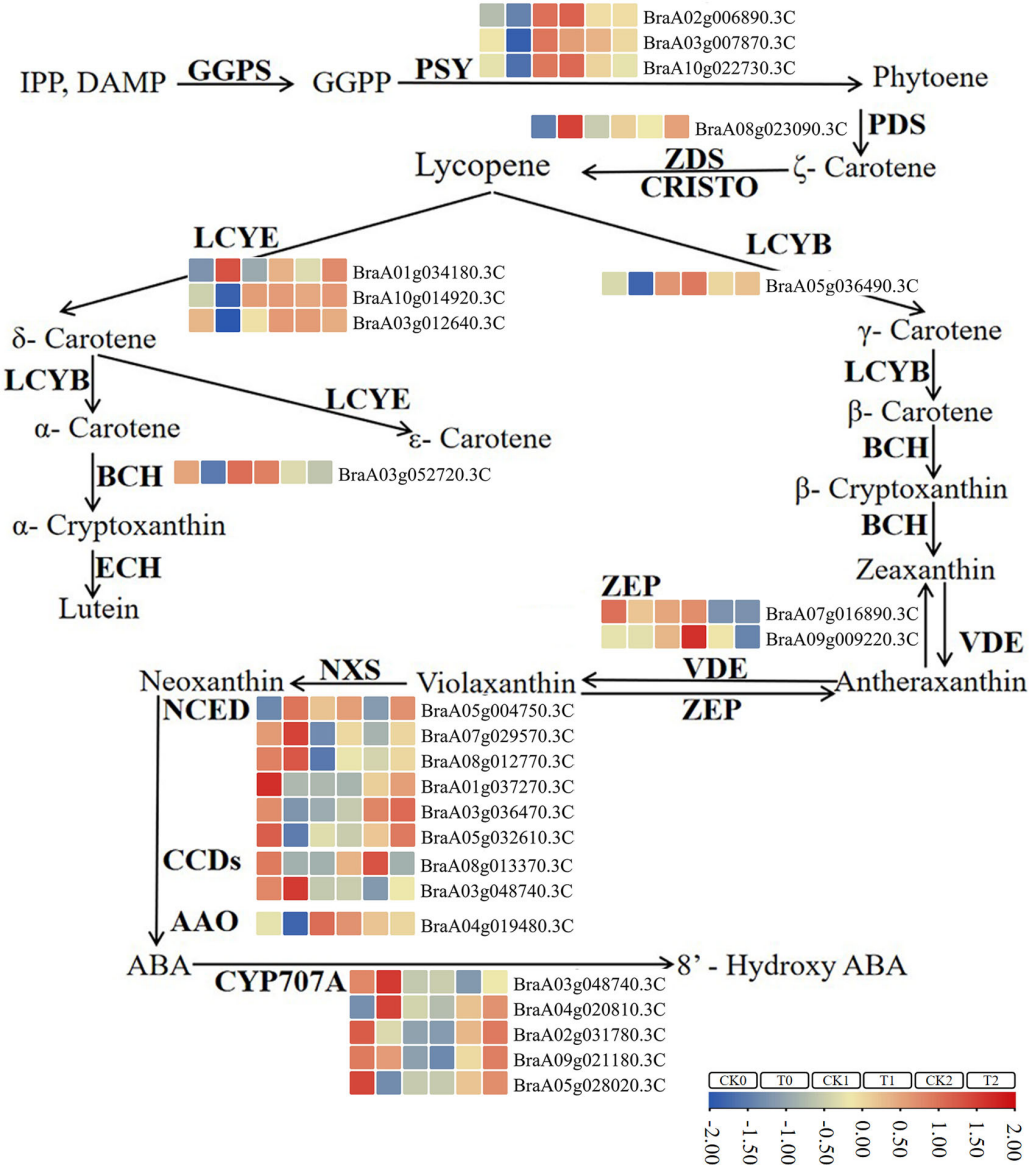
The identified DEGs involved in the pathway of carotenoid biosynthesis in 19GC-2 and 19YC-2. CK0, T0, CK1, T1, CK2, and T2 represent the six cDNA libraries. The boxes in different colors indicate gene expression in corresponding library. Gene expression levels are based on log2 FPKM values.

The pathway enrichment analysis of DEGs indicated that carotenoid metabolic pathway may be associated with the leaf color differences in 19GC-2 and 19YC-2 under different light intensity treatment. As expected, a total of 25 DEGs were identified and involved in carotenoid biosynthesis (**Figure 7**; **Supplementary Table S5**). Among these DEGs, six upregulated and 16 downregulated genes were found in the comparison group of CK0 vs T0, nine upregulated genes were found in the comparison group of CK1 vs T1, and five upregulated and three downregulated genes were found in the comparison group of CK2 vs T2 (**Supplementary Table S5**). Moreover, two genes of *BrPDS* (*BraA08g023090.3C*) and *BrLCYE* (*BraA01g034180.3C*) were significant upregulated expression in the comparison groups of CK0 vs T0 and CK2 vs T2, and three genes of *BrAAO3* (*BraA04g019480.3C*), *BrCCD4* (*BraA01g010560.3C*), and *BrNCED3* (*BraA05g032610.3C*) were significant downregulated expression in the comparison groups of CK0 vs T0 and CK2 vs T2.

**Figure 8:**
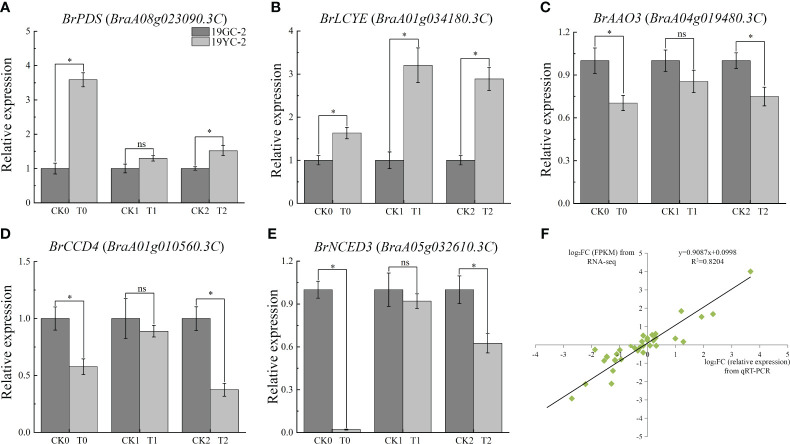
Expression analysis of critical carotenoid biosynthetic DEGs by qRT-PCR in 19GC-2 and 19YC-2. **(A)** *BrPDS* gene. **(B)** *BrLCYE* gene. **(C)** *BrAAO3* gene. **(D)** *BrCCD4* gene. **(E)** *BrNCED3* gene. **(F)** Correlation between qRT-PCR and RNA-seq analysis. CK0, T0, CK1, T1, CK2, and T2 represent the six cDNA libraries from 19GC-2 and 19YC-2. ns, no significant; *P < 0.05.

Further qRT-PCR analysis showed that the expression levels of *BrPDS* and *BrLCYE* genes that were the upstream genes of carotenoid biosynthesis, were increased in the three comparison groups and showed significant increases in CK0 vs T0 and CK2 vs T2 (**Figures 8A, B**). The expression levels of *BrAAO3*, *BrCCD4*, and *BrNCED3* genes that were the downstream genes of carotenoid biosynthesis, were significantly decreased in CK0 vs T0 and CK2 vs T2 (**Figures 8C-E**). The results indicated that the upregulation of upstream genes and the downregulation of downstream genes were accordant with the changes in carotenoid contents. In addition, the results of qRT-PCR analysis in gene expressions were almost identical to the results of RNA-seq analysis (*R^2^ =* 0.7784) (**Figure 8F**), demonstrating the reliability of RNA-seq data.

### 3.8 Analysis of co-expression network of DEGs and identification of transcription factors involved in carotenoid metabolism

**Figure 9:**
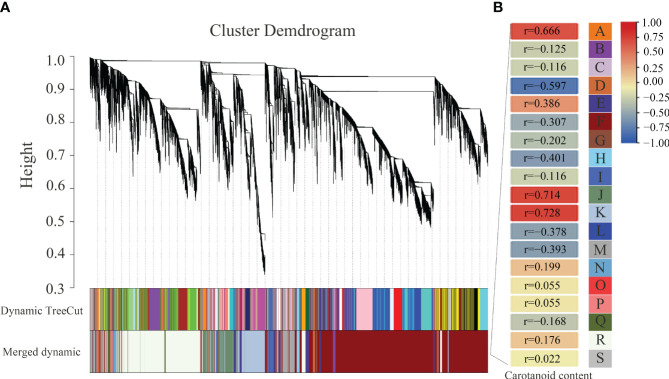
Association analysis between DEGs and carotenoid contents by weighted gene co-expression network analysis (WGCNA) in 19GC-2 and 19YC-2. **(A)** The hierarchical cluster tree of correlated modules. **(B)** The identified highly correlated modules represented by different letters from A to S.

To further investigate the relationships of gene differential expression and carotenoid content, WGCNA was performed to identify the highly correlated modules of DEGs. As a result, 19 modules named from A to S and labeled by different colors were identified (**Figure 9**). The A (r = 0.666), J (r = 0.714), and K (r = 0.728) modules containing 97, 1371, and 1828 DEGs, respectively, were highly positively related to the changes in carotenoid content, and the D (r = 0.597) module containing 175 DEGs was highly negatively related to the changes in carotenoid content. In addition, the upregulated expressed *BrPDS* and *BrLCYE* genes were assigned to the positively related J module, which was consistent with the positive regulation of the two genes in carotenoid synthesis. The downregulated expressed *BrAAO3* and *BrCCD4* were assigned to the negatively related F (r=−0.307) module, and the downregulated expressed *BrNCED3* was assigned to the negatively related M (r=−0.393) module. The downregulated expressed *BrCAB1* was assigned to the negatively related F (r=−0.307) module, and the downregulated expressed *BrCAB13*, *BrCAB36*, *BrLHCB1.3* and *BrLHCB4.2* were assigned to the negatively related M (r=−0.393) module. The results suggest that these DEGs identified in these highly correlated modules may play potential roles in regulating the pathway of carotenoid biosynthesis and metabolism.

**Figure 10:**
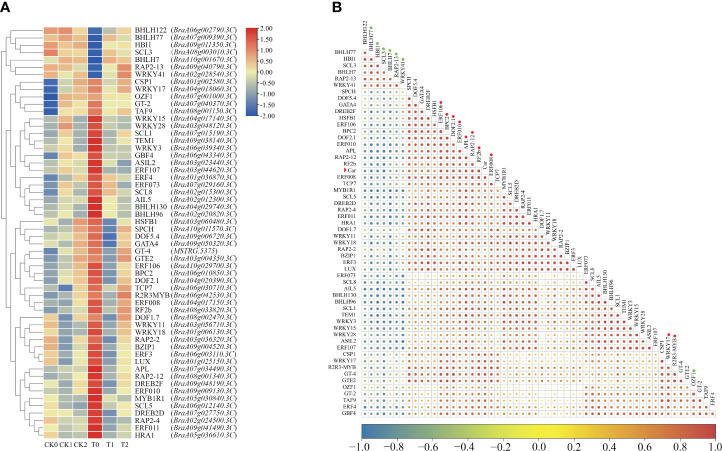
Identification of differentially expressed TFs related to carotenoid metabolism by WGCNA. **(A)** Heat map of differentially expressed TFs. **(B)** Correlation of TFs and carotenoid content. The red arrowhead indicates the carotenoid (car). The circles after gene names indicate the strongly positively (in red) and negatively (in green) correlated TFs.

Furthermore, a total of 56 DEGs belonging to transcription factors (TFs) were identified in these highly correlated modules by WGCNA (**Figure 10**). Among these differentially expressed TFs, most of these TFs were upregulated expression in the comparison group of CK0 vs T0, and seven genes were downregulated expression. Correlation analysis showed that the differential expressions of 17 TFs were strongly correlated with the changes in carotenoid contents (**Figure 10B**; **Supplementary Table S6**). For instance, the gene expression of *BraA08g033820.3C* (*BrRF2b*), *BraA08g001340.3C* (*BrRAP2-12*), *BraA04g017150.3C* (*BrERF008*), and *BraA06g042530.3C* (*BrR2R3-MYB*) had highly positive correlation (r > 0.91) to the changes in carotenoid contents, and the gene expression of *BraA08g003010.3C* (*BrSCL3*) and *BraA07g009390.3C* (*BrbHLH77*) had highly negative correlation (r < −0.9) to the changes in carotenoid contents. Moreover, many genes were identified as the transcription factor families of *BrERF/AP2* (*BraA08g001340.3C*, *BraA04g017150.3C*, *BraA10g029700.3C*, *BraA09g009130.3C*, and *BraA09g040790.3C*), *BrR2R3-MYB* (*BraA06g042530.3C*), *BrbHLH* (*BraA10g001670.3C*, *BraA09g011350.3C*, and *BraA07g009390.3C*), and *BrWRKY* (*BraA04g018060.3C* and *BraA02g028540.3C*) (**Supplementary Table S6**). In general, these identified DEGs and TFs that highly related to carotenoid biosynthesis and metabolism may play important roles in regulating leaf color changes in Chinese cabbage yellow cotyledon mutant 19YC-2.

## 4 Discussion

### 4.1 Changes in carotenoid content may determine the leaf color changes in Chinese cabbage yellow cotyledon mutant in response to different light intensities

Leaf color formation is closely related to the contents of photosynthetic pigments (Terry et al., 2001). Leaf color mutants are easy to recognize in leaf phenotypes and often used as valuable materials for investigating pigment biosynthesis and enriching the germplasms in genetic breeding (Deng et al., 2012). Various leaf color mutants usually impact the accumulation and metabolism of photosynthetic pigments and have been characterized in many horticultural crops (Wang et al., 2004; Zhao et al., 2020). In this study, the Chinese cabbage yellow cotyledon mutant 19YC-2 is a distinctive mutant that displays obvious yellow phenotype in the cotyledons. Like the other reported yellow leaf mutants, the Chinese cabbage yellow cotyledon mutant has special excellent characters and can be used as a selective biomarker for hybrid breeding. More importantly, yellow cotyledon phenotype can be directly selected at the earlier cotyledon stage for hybrid purity identification, which shortens the breeding time and improves the breeding efficiency.

Most yellow leaf phenotype of mutant is mainly determined by the changed contents in chlorophyll and carotenoid and the disrupted chloroplast development (Sakamoto, 2003; Cheng et al., 2022). Previous study reported the reduced chlorophyll content in the yellow leaf mutant of ginkgo (*Ginkgo biloba*) (Sun et al., 2022). In the green leaf mutant of *Populus deltoides* Marsh, the leaf color was associated with the lower levels of chlorophyll and carotenoid (Zhang et al., 2019). The yellow-green leaf color in wheat (*Triticum aestivum L.*) was consequence of the decreased chlorophyll content and abnormal chloroplast development (Wu et al., 2018). In addition, chlorophyll deficient was also showed to cause yellow leaf phenotypes in watermelon (Xu et al., 2023), and the chlorophyll deficient mutant in Pak-choi exhibited the obvious yellow leaves and the reduced total chlorophyll content (Zhang et al., 2017). As expected, our studies also showed the changes in chlorophyll and carotenoid contents in Chinese cabbage yellow cotyledon mutant 19YC-2. The significantly higher carotenoid content and lower chlorophyll content were found in the cotyledons of 19YC-2, which may be responsible for the yellow cotyledon phenotypes and was similar to the previous reports (Zhang et al., 2015; Sun et al., 2022; Xu et al., 2023). It is known that chloroplast development also affects the accumulation of chlorophyll and carotenoid and leaf color changes (Pogson and Albrecht, 2011; Zhao et al., 2020). The thylakoid is an important part of chloroplast structures and essential for chloroplast function (Allen and Forsberg, 2001; Pogson and Albrecht, 2011). Many studies in leaf color mutants demonstrated that the variations in thylakoid structure caused the decreases in grana numbers and thylakoid lamella, resulting in the damaged chloroplast ultrastructure (Nie et al., 2021; Wang et al., 2022; Zhang et al., 2022b). In the current study, observation of leaf color found that the 1^st^ leaves of 19YC-2 gradually turned green with the growth of seedlings. Observation of chloroplast ultrastructure showed that the cotyledons and the yellow 1^st^ leaves of 19YC-2 exhibited the abnormal chloroplast and thylakoid structures with the absence of grana lamellae and starch grains, while the normal chloroplast in shape and structure were observed in the turn-green 1^st^ leaves of 19YC-2 at the four-leaf stage. The abnormal chloroplast ultrastructure may contribute to the yellow phenotypes in cotyledons and leaves of 19YC-2 mutant, which was consistent with many other reports (Nie et al., 2021; Wang et al., 2022; Zhang et al., 2022b). The results suggest that the changes in the contents of photosynthetic pigments in yellow cotyledon mutant 19YC-2 may be attributed to the abnormal chloroplast development, which can impair photosynthetic efficiency (Pogson and Albrecht, 2011).

Numerous environmental factors, such as light, have been described to affect the development and biogenesis of chloroplast *via* photomorphogenic pathways (Xu et al., 2021; Chen et al., 2023b). Long-term deficient light can lead to degradation of chlorophyll and leaf yellowing (Lee et al., 2014). Different light intensities can affect the chloroplast formation and the synthesis and degradation of photosynthetic pigments (Kong et al., 2016). The mutants of lacking photosynthetic electron transport in *Arabidopsis* exhibited a variegated phenotype under high light intensity and a green phenotype under low light intensity (Rosso et al., 2009). The yellow leaf mutant in pepper showed the increase in carotenoid content under high light intensity (Liu et al., 2023). Our previous study also found that low-intensity light treatment caused the turn-green leaves in Chinese cabbage yellow cotyledon mutant 19YC-2. Therefore, to investigate the underlying physiological mechanism of leaf color change in 19YC-2 under low-intensity light treatment is significant in this study. We found that the leaves of 19YC-2 showed higher carotenoid and lower chlorophyll contents under normal light condition than that under low-intensity light condition. The observation was coincident with that the leaves of 19YC-2 exhibited yellow exposed to normal light but turned green under low-intensity light. These results indicated that low-intensity light treatment primarily changed the carotenoid and chlorophyll contents and then induced the transition from yellow to green in the new leaves of 19YC-2. Meanwhile, RNA-seq analysis and KEGG pathway enrichment revealed that many DEGs were identified in the significantly enriched pathway of carotenoid biosynthesis in CK0 vs T0 (the cotyledon stage) and CK2 vs T2 (normal light treatment), but not in CK1 vs T1 (low-intensity light treatment), which indicated that the carotenoid biosynthesis and metabolism may be closely related to leaf color changes in 19YC-2. However, the pathway related to porphyrin and chlorophyll metabolism was enriched only in CK0 vs T0, but not in CK1 vs T1 and CK2 vs T2, implying that different light intensity treatments may have no significant effects on the DEGs related to chlorophyll biosynthesis in yellow cotyledon mutant 19YC-2. In addition, the existence of photosynthetic antenna proteins can increase the rate of energy delivery and photosynthetic efficiency (Pogson and Albrecht, 2011; Ruban, 2015). In the current study, the expression levels of DEGs encoding photosynthesis antenna proteins, such as *BrCAB1*, *BrCAB13*, *BrCAB36*, *BrLHCB1.3*, and *BrLHCB4.2*, were significantly decreased in CK0 vs T0 and CK2 vs T2, suggesting the suppressed photosynthesis in the yellow cotyledons and yellow leaves in 19YC-2.

### 4.2 The putative regulatory pathway of carotenoid biosynthesis in Chinese cabbage yellow cotyledon mutant

Carotenoid biosynthesis is documented to be influenced by the changes in light and is stalled in darkness (Yuan et al., 2015; Xu et al., 2021). Carotenoids are synthesized in plastids, which is regulated by the complex and highly conserved biosynthetic pathway that has been well established and involves the transcription of many biosynthetic genes (Nisar et al., 2015; Yuan et al., 2015). Among these key genes that encode carotenogenic enzymes, *PDS* and *ζ*-carotene desaturase (*ZDS*) genes are responsible for the conversion of uncolored phytoene to red-colored lycopene and have been identified and studied in many colored fruits and crops (Fanciullino et al., 2007; Yuan et al., 2015). The high expression of *PDS* gene had been found to be correlated with the accumulation of carotenoid during tomato fruit ripening and citrus fruit development (Pecker et al., 1992; Kato et al., 2004; Rodrigo et al., 2004). The subsequent two biochemical pathways, lycopene converted to lutein and zeaxanthin, were mediated by *LCYE* and *LCYB* genes (Nisar et al., 2015). These carotenoid pigments are abundant not only in the yellow fruits and flowers, but also in leafy vegetables exposed to light (Yuan et al., 2015). Studies showed that *LCYE* mutation caused lutein deficiency and higher *β*-carotene accumulation (Pogson et al., 1996), and the high expression of *LCYE* gene promoted lutein accumulation in tomato and strawberry (Zhu et al., 2015; Yuan et al., 2022). Exploring the expression changes of these carotenoid biosynthesis related genes and their potential function can contribute to further understand the mechanism of leaf color changes in Chinese cabbage yellow cotyledon mutant 19YC-2. In this study, RNA-seq analysis showed that *BrPDS* (*BraA08g023090.3C*) and *BrLCYE* (*BraA01g034180.3C*) genes were upregulated expression in the yellow leaves of yellow cotyledon mutant 19YC-2, indicating the putative regulatory roles of *BrPDS* and *BrLCYE* in leaf color changes in 19YC-2.

Furthermore, the downstream genes in the pathway of carotenoid metabolism, such as *BrAAO3* (*BraA04g019480.3C*), *BrCCD4* (*BraA01g010560.3C*), and *BrNCED3* (*BraA05g032610.3C*), were found to be downregulated expression in the cotyledons and leaves of 19YC-2 when displayed yellow. In the downstream process of carotenoid metabolism, a list of enzymes can cleave carotenoid to produce phytohormone abscisic acid (ABA) and strigolactones, and the downregulated expression of these downstream genes had been extensively demonstrated to be involved in the catabolism of carotenoid (Nisar et al., 2015; Yuan et al., 2015). The expression of *NCED* gene in ginkgo was increased in yellow leaves in autumn, while decreased in green leaves in summer (Sun et al., 2022). Previous studies in zucchini reported that *NCED* and *CCD* genes were downregulated in yellow and orange peels, which was agreement with the accumulation of yellow lutein (Obrero et al., 2013; Niu et al., 2023). These evidences suggest that the differential expression of carotenoid-related genes combining the alteration of carotenoid content favor the leaf color changes in yellow cotyledon mutant 19YC-2.

Many putative regulators have been identified to function in transcriptional regulation of carotenoid biosynthetic genes in photosynthetic tissues, which is highly influenced by various environmental factors (Stanley and Yuan, 2019). In the current study, many differentially expressed TFs were identified to be highly correlated with the changes in carotenoid contents by WGCNA, and belonged to the families of *BrERF/AP2*, *BrR2R3-MYB*, *BrbHLH*, and *BrWRKY*. The *BrERF/AP2* family contains many TFs that had been characterized to participate in regulating the process of carotenoid metabolism (Yuan et al., 2015; Stanley and Yuan, 2019). In *Arabidopsis* leaves, *RAP2.2* (a member of *ERF/AP2* family) was reported to interact with *SINAT2* and positively regulate *PSY* and *PDS* during carotenoid biosynthesis (Welsch et al., 2007). In tomato, an *AP2a* gene was found to be a major regulator of the upstream genes of carotenoid biosynthesis, mediating fruit ripening (Chung et al., 2010; Karlova et al., 2011). We also found that two DEGs, *BraA08g001340.3C* and *BraA04g017150.3C*, were identified to *BrERF/AP2* family and highly positively related to the changes in carotenoid contents, which provide important references for exploring the functions of the two TFs. Meanwhile, the TFs of *MYB* family had been demonstrated to involve in the transcriptional regulation of leaf-color mutation in ginkgo (Wu et al., 2019). In kiwifruit, *MYB7* gene was found to activate the promoter of *LCYB* gene that modulates carotenoid accumulation (Ampomah-Dwamena et al., 2019). In the current study, a *BrMYB* gene (*BraA06g042530.3C*) was upregulated expressed in the cotyledons and leaves of yellow cotyledon mutant 19YC-2 and highly positively related to carotenoid accumulation. Overall, these differentially expressed TF genes may play potential important roles in regulating carotenoid biosynthesis mediating leaf color change in yellow cotyledon mutant 19YC-2.

## 5 Conclusion

**Figure 11:**
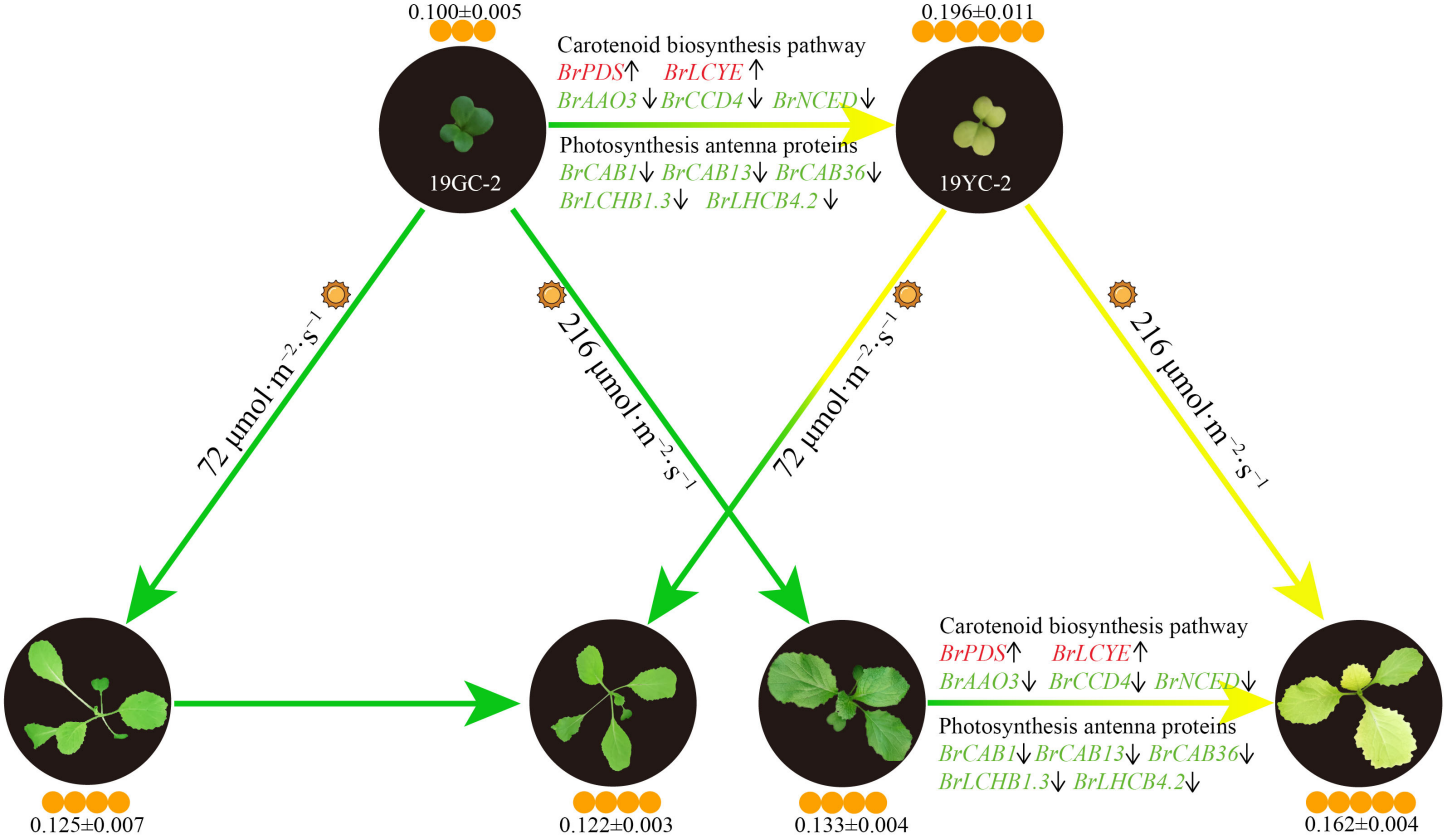
Schematic diagram of the regulatory network of leaf color changes of Chinese cabbage yellow cotyledon mutants under different light intensity. The color of gene name indicates the upregulation (red) or downregulation (green) of gene expression, and the arrows after gene names indicate increases (upward) or decreases (downward) in gene expression levels. The sun represents light conditions. The number of orange circles indicates the relative content of carotenoids (± SEM; mg·g^−1^/FW).

Chinese cabbage yellow cotyledon mutant 19YC-2 has obvious yellow phenotypes in the cotyledons and the new leaves, which is valuable for hybrid breeding. In this study, we found that low-intensity light treatment induced an obvious change from yellow to green in the new leaves of 19YC-2. The decreased carotenoid content may be the major reason of leaf color change in 19YC-2 under low-intensity light treatment. RNA-seq analysis identified that the expression changes of many DEGs encoding photosynthesis antenna proteins and related to carotenoid biosynthesis were consistent with the changes in carotenoid content, indicating the putative important roles of these DEGs in regulating leaf color changes of 19YC-2. Based on the above results, we drew a schematic diagram on the regulatory network of leaf color changes of Chinese cabbage yellow cotyledon mutants exposed to different light intensity (**Figure 11**). These results facilitate understanding the effects of photosynthetic pigments on leaf color changes and provide insights into the formation mechanisms of yellow cotyledons and yellow leaves in Chinese cabbage 19YC-2.

## Data availability statement

The datasets presented in this study can be found in online repositories. The names of the repository/repositories and accession number(s) can be found in the article/**Supplementary Material**.

## Author contributions

JH: Data curation, Formal analysis, Investigation, Methodology, Software, Validation, Writing – original draft. NZ: Data curation, Formal analysis, Investigation, Validation, Writing – original draft. YG: Investigation, Validation, Writing – original draft. YB: Investigation, Validation, Writing – original draft. YL: Investigation, Validation, Writing – original draft. LZ: Conceptualization, Funding acquisition, Methodology, Resources, Supervision, Writing – review & editing. SN: Conceptualization, Data curation, Funding acquisition, Methodology, Supervision, Writing – original draft, Writing – review & editing.

## Supplementary material

Supplementary Figure 1 Enrichment analysis of the identified DEGs based on GO terms. CK0, T0, CK1, T1, CK2, and T2 represent the six cDNA libraries from 19GC-2 and 19YC-2.

Supplementary Figure 2 Top 30 enriched KEGG pathways of the identified DEGs. CK0, T0, CK1, T1, CK2, and T2 represent the six cDNA libraries from 19GC-2 and 19YC-2. Red circles indicate the critical pathways.

Supplementary Figure 3 GSEA analysis of the pathways of photosynthesis antenna proteins **(A)** and carotenoid biosynthesis **(B)**. CK0, T0, CK1, T1, CK2, and T2 represent the six cDNA libraries from 19GC-2 and 19YC-2.
