# Supplementary material for: Effects of different light intensity on leaf color changes in a Chinese cabbage yellow cotyledon mutant
Source: Front Plant Sci. 2024 Apr 16;15:1371451. doi: 10.3389/fpls.2024.1371451 (PMC11058996; doi:10.3389/fpls.2024.1371451)
Supplement: Supplementary file 1 [file Table_1.docx]

**Supplementary** **Table S1** Specific primer sequences used for qRT-PCR analysis.

| **Gene** | **Primer sequence (5'-3') forward** | **Primer sequence (5'-3') reverse** |
| --- | --- | --- |
| *BrCAB1* | AGAACGGAAGGTTGGCTATG | AGGGACGAAGTTGGTGGC |
| *BrCAB13* | TGGGCAATGTTGGGAGC | CGTGGACAAGGTTTGGGTT |
| *BrCAB36* | AACGGTCGGCTTGCTATG | GGGACGAAGTTGGTGGC |
| *BrLHCB1.3* | GCCGAGTTGAAAGTGAAGGA | ATGCGTTGTTGTTGACTGGAT |
| *BrLHCB4.2* | ACCAGAACTTAGCCAAGAACATA | TGGAGCCCAAAGACCTCA |
| *BrPDS* | TTGAGTCCGATAGTCACTACAT | TACTTGCTGCCTCTACCCT |
| *BrLCYE* | AAATGAATAGCCTGTTGCG | AATCGGTGGTTCCTTGC |
| *BrNCED3* | AAAATACTTCCGCTTCTCACC | CGCGACCTTCTCCTTGTC |
| *BrCCD4* | ATCAATCCGGCGTTCGTT | GCAATCGTCCCTATCTCCTCTA |
| *BrAAO3* | GATGAAGACAACTGCGTGAA | GCCAAAGCCACCTCCAACT |
| *BrEF-1α* | GCCAAAGAGGCCATCAGACAA | ATACCAGGCTTGAGCATACCG |

**Supplementary** **Table S2** Summary of RNA-seq data in Chinese cabbage 19GC-2 and 19YC-2.

| Sample | Raw Data | Clean Data (%) | Q30 (%) | GC content (%) |
| --- | --- | --- | --- | --- |
| CK0-1 | 44270268 | 44079208 (99.57%) | 6112705313 (93.01%) | 3194074862 (48.60%) |
| CK0-2 | 36711580 | 36541442 (99.54%) | 5114230965 (93.72%) | 2626681832 (48.14%) |
| CK0-3 | 40707962 | 40527976 (99.56%) | 5632844209 (92.99%) | 2946153716 (48.64%) |
| CK1-1 | 39332696 | 39149414 (99.53%) | 5441319506 (93.14%) | 2750204756 (47.07%) |
| CK1-2 | 36836236 | 36659914 (99.52%) | 5118395128 (93.45%) | 2581027028 (47.12%) |
| CK1-3 | 44208184 | 43995958 (99.52%) | 6119503232 (93.27%) | 3104142036 (47.31%) |
| CK2-1 | 40911004 | 40685902 (99.45%) | 5666835283 (93.36%) | 2894143988 (47.68%) |
| CK2-2 | 48156894 | 47909408 (99.49%) | 6702372424 (93.79%) | 3407209192 (47.68%) |
| CK2-3 | 37539218 | 37371392 (99.55%) | 5190052913 (93.23%) | 2645371685 (47.52%) |
| T0-1 | 43112366 | 42915520 (99.54%) | 5948524381 (93.05%) | 3061243197 (47.88%) |
| T0-2 | 39591016 | 39399958 (99.52%) | 5463656181 (93.13%) | 2814286163 (47.97%) |
| T0-3 | 40989942 | 40810100 (99.56%) | 5696757465 (93.60%) | 2929423892 (48.13%) |
| T1-1 | 41026736 | 40843790 (99.55%) | 5699053812 (93.52%) | 2894164202 (47.49%) |
| T1-2 | 48344138 | 48113442 (99.52%) | 6644305198 (92.63%) | 3402372316 (47.44%) |
| T1-3 | 43314232 | 43104038 (99.51%) | 5938295887 (92.40%) | 3052129291 (47.49%) |
| T2-1 | 41370038 | 41189590 (99.56%) | 5732245577 (93.46%) | 2921174268 (47.63%) |
| T2-2 | 36466840 | 36317368 (99.59%) | 5027673282 (92.85%) | 2582191094 (47.69%) |
| T2-3 | 39158872 | 39008864 (99.62%) | 5417525639 (93.04%) | 2771038274 (47.59%) |

**Supplementary** **Table S3** Statistics of total reads mapped to the reference genome of Chinese cabbage.

| Sample | Total Read | Unmapped (%) | Total Mapped (%) |
| --- | --- | --- | --- |
| CK0-1 | 43854130 | 3133408 (7.15%) | 40720722 (92.85%) |
| CK0-2 | 36339638 | 2589533 (7.13%) | 33750105 (92.87%) |
| CK0-3 | 40294452 | 2973915 (7.38%) | 37320537 (92.62%) |
| CK1-1 | 39013740 | 3138892 (8.05%) | 35874848 (91.95%) |
| CK1-2 | 36508802 | 2886124 (7.91%) | 33622678 (92.09%) |
| CK1-3 | 43823788 | 3475608 (7.93%) | 40348180 (92.07%) |
| CK2-1 | 40394750 | 3283029 (8.13%) | 37111721 (91.87%) |
| CK2-2 | 47532706 | 3709022 (7.80%) | 43823684 (92.20%) |
| CK2-3 | 37242548 | 3081179 (8.27%) | 34161369 (91.73%) |
| T0-1 | 42680806 | 3190358 (7.47%) | 39490448 (92.53%) |
| T0-2 | 38735148 | 2897365 (7.48%) | 35837783 (92.52%) |
| T0-3 | 40151328 | 2927994 (7.29%) | 37223334 (92.71%) |
| T1-1 | 40527554 | 3100157 (7.65%) | 37427397 (92.35%) |
| T1-2 | 47904714 | 3900391 (8.14%) | 44004323 (91.86%) |
| T1-3 | 42903934 | 3440531 (8.02%) | 39463403 (91.98%) |
| T2-1 | 40882406 | 3351127 (8.20%) | 37531279 (91.80%) |
| T2-2 | 36209020 | 3005864 (8.30%) | 33203156 (91.70%) |
| T2-3 | 38861750 | 3207311 (8.25%) | 35654439 (91.75%) |

**Supplementary** **Table S4** Information of DEGs related to photosynthesis antenna proteins.

| Gene ID | Gene name | CK0 FPKM | T0 FPKM | Significant T0/CK0 | CK1 FPKM | T1 FPKM | Significant T1/CK1 | CK2 FPKM | T2 FPKM | Significant T2/CK2 |
| --- | --- | --- | --- | --- | --- | --- | --- | --- | --- | --- |
| *BraA02g037860.3C* | *BrCAB36* | 7009.957 | 1077.676 | DOWN | 224.023 | 201.254 | ns | 49.97 | 18.348 | DOWN |
| *BraA03g018080.3C* | *BrCAB1* | 13063.183 | 5850.009 | DOWN | 711.778 | 756.036 | ns | 243.797 | 108.948 | DOWN |
| *BraA03g027230.3C* | *BrLHCB5* | 42.214 | 18.457 | DOWN | 22.317 | 21.676 | ns | 12.424 | 5.406 | ns |
| *BraA03g041940.3C* | *BrCAB-151* | 4500.458 | 1018.488 | DOWN | 33.023 | 27.095 | ns | 30.479 | 9.445 | ns |
| *BraA04g024630.3C* | *BrLHCB1.3* | 28068.701 | 15132.225 | ns | 2041.593 | 2157.032 | ns | 1525.741 | 451.472 | DOWN |
| *BraA05g010690.3C* | *BrCAB1* | 8345.138 | 2586.023 | DOWN | 26.141 | 18.142 | ns | 45.419 | 8.404 | ns |
| *BraA05g010700.3C* | *BrCAB1* | 5390.711 | 1523.014 | DOWN | 32.644 | 68.7 | ns | 73.094 | 35.647 | ns |
| *BraA05g037360.3C* | *BrLHCB4.2* | 5302.078 | 2173.791 | DOWN | 551.768 | 547.248 | ns | 575.824 | 271.599 | DOWN |
| *BraA06g011900.3C* | *BrCAP10B* | 3473.6 | 1289.893 | DOWN | 249.363 | 311.501 | ns | 417.495 | 227.918 | ns |
| *BraA06g019540.3C* | *BrLHCA4* | 1196.441 | 717.584 | ns | 184.323 | 196.483 | ns | 156.734 | 70.733 | DOWN |
| *BraA06g037410.3C* | *BrCAB36* | 4803.498 | 574.734 | DOWN | 71.69 | 82.635 | ns | 12.941 | 6.424 | ns |
| *BraA07g010770.3C* | *BrLHCB1.3* | 15550.587 | 4947.626 | DOWN | 2378.578 | 2947.729 | ns | 1672.028 | 788.613 | DOWN |
| *BraA07g010780.3C* | *BrLHCB1.3* | 15550.587 | 4947.626 | DOWN | 2378.578 | 2947.729 | ns | 1672.028 | 788.613 | DOWN |
| *BraA08g023650.3C* | *BrLHCB1.3* | 31410.649 | 15535.59 | DOWN | 5913.179 | 5947.141 | ns | 4627.7 | 3543.507 | ns |
| *BraA09g003290.3C* | *BrCAB36* | 5185.865 | 1736.148 | DOWN | 188.212 | 189.795 | ns | 70.724 | 14.124 | ns |
| *BraA09g058520.3C* | *BrCAP10A* | 2075.995 | 625.188 | DOWN | 32.83 | 31.89 | ns | 75.973 | 56.24 | ns |
| *BraA10g012410.3C* | *BrCAB13* | 5085.896 | 1096.754 | DOWN | 40.907 | 47.309 | ns | 42.639 | 0.072 | DOWN |

Note: UP: upregulated expression; DOWM: downregulated expression; ns: no significant.

**Supplementary** **Table S5** Information of DEGs related to the pathway of carotenoid biosynthesis.

| Gene ID | Gene name | CK0 FPKM | T0 FPKM | Significant T0/CK0 | CK1 FPKM | T1 FPKM | Significant T1/CK1 | CK2 FPKM | T2 FPKM | Significant T2/CK2 |
| --- | --- | --- | --- | --- | --- | --- | --- | --- | --- | --- |
| *BraA04g019480.3C* | *BrAAO3* | 1.284 | 0.469 | DOWN | 2.967 | 2.334 | ns | 1.704 | 1.530 | DOWN |
| *BraA03g052720.3C* | *BrBCH* | 6.480 | 2.215 | DOWN | 8.623 | 7.817 | ns | 4.161 | 3.620 | ns |
| *BraA01g010560.3C* | *BrCCD4* | 62.482 | 32.533 | DOWN | 254.317 | 185.084 | ns | 25.220 | 10.723 | DOWN |
| *BraA08g013370.3C* | *BrCCD4* | 0.142 | 0.001 | ns | 0.001 | 0.031 | ns | 0.409 | 0.001 | DOWN |
| *BraA03g048740.3C* | *BrCYP707A1* | 11.418 | 45.360 | UP | 0.929 | 1.039 | ns | 0.308 | 2.027 | UP |
| *BraA04g020810.3C* | *BrCYP707A2* | 0.105 | 5.658 | UP | 0.364 | 0.245 | ns | 0.944 | 1.870 | UP |
| *BraA02g031780.3C* | *BrCYP707A3* | 122.569 | 13.685 | DOWN | 5.260 | 4.633 | ns | 35.895 | 81.515 | ns |
| *BraA09g021180.3C* | *BrCYP707A3* | 101.676 | 59.911 | ns | 3.918 | 2.480 | ns | 22.391 | 94.443 | UP |
| *BraA05g028020.3C* | *BrCYP707A4* | 0.497 | 0.056 | DOWN | 0.104 | 0.104 | ns | 0.188 | 0.285 | ns |
| *BraA05g036490.3C* | *BrLCYB* | 68.508 | 31.976 | DOWN | 118.051 | 136.305 | UP | 84.450 | 94.643 | ns |
| *BraA01g034180.3C* | *BrLCYE* | 0.025 | 0.550 | UP | 0.035 | 0.175 | UP | 0.074 | 0.280 | UP |
| *BraA10g014920.3C* | *BrLCYE* | 2.510 | 1.012 | DOWN | 4.981 | 5.048 | ns | 4.659 | 5.149 | ns |
| *BraA03g012640.3C* | *BrLCYE* | 5.929 | 2.429 | DOWN | 5.065 | 6.597 | UP | 6.549 | 6.145 | ns |
| *BraA05g004750.3C* | *BrNCED* | 0.001 | 1.487 | UP | 0.140 | 0.414 | ns | 0.002 | 0.626 | ns |
| *BraA07g029570.3C* | *BrNCED* | 0.768 | 2.051 | UP | 0.081 | 0.386 | UP | 0.145 | 0.396 | ns |
| *BraA08g012770.3C* | *BrNCED* | 2.043 | 3.643 | ns | 0.069 | 0.456 | UP | 0.344 | 0.637 | ns |
| *BraA01g037270.3C* | *BrNCED3* | 28.246 | 2.405 | DOWN | 2.393 | 2.302 | ns | 5.817 | 9.326 | ns |
| *BraA03g036470.3C* | *BrNCED3* | 11.581 | 0.707 | DOWN | 1.059 | 1.847 | ns | 12.810 | 18.414 | ns |
| *BraA05g032610.3C* | *BrNCED3* | 19.428 | 1.619 | DOWN | 4.848 | 4.047 | ns | 15.284 | 7.692 | DOWN |
| *BraA08g023090.3C* | *BrPDS* | 2.762 | 6.360 | UP | 3.631 | 4.327 | UP | 4.028 | 4.908 | UP |
| *BraA02g006890.3C* | *BrPSY1* | 11.997 | 4.464 | DOWN | 125.980 | 152.039 | UP | 29.278 | 29.772 | ns |
| *BraA03g007870.3C* | *BrPSY1* | 10.690 | 4.737 | DOWN | 17.495 | 14.708 | ns | 13.340 | 11.279 | ns |
| *BraA10g022730.3C* | *BrPSY1* | 61.526 | 15.833 | DOWN | 197.471 | 226.847 | UP | 83.349 | 62.015 | ns |
| *BraA07g016890.3C* | *BrZEP* | 15.638 | 10.306 | DOWN | 12.014 | 13.474 | ns | 5.058 | 5.028 | ns |
| *BraA09g009220.3C* | *BrZEP* | 0.210 | 0.203 | DOWN | 0.355 | 1.144 | UP | 0.233 | 0.072 | ns |

Note: UP: upregulated expression; DOWM: downregulated expression; ns: no significant.

**Supplementary** **Table S6** The differentially expressed TFs highly related to carotenoid contents by WGCNA.

| Gene ID | Gene name | r value | TF family |
| --- | --- | --- | --- |
| *BraA08g033820.3C* | *BrRF2b* | 0.99 | *bZIP* |
| *BraA08g001340.3C* | *BrRAP2-12* | 0.95 | *ERF/AP2* |
| *BraA04g017150.3C* | *BrERF008* | 0.94 | *ERF/AP2* |
| *BraA06g042530.3C* | *BrR2R3-MYB* | 0.92 | *R2R3-MYB* |
| *BraA10g029700.3C* | *BrERF106* | 0.91 | *ERF/AP2* |
| *BraA07g034490.3C* | *BrAPL* | 0.9 | *OZF* |
| *BraA06g010850.3C* | *BrBPC2* | 0.9 | *BPC* |
| *BraA04g020390.3C* | *BrDOF2.1* | 0.9 | *DOF* |
| *BraA09g009130.3C* | *BrERF010* | 0.87 | *ERF/AP2* |
| *BraA04g018060.3C* | *BrWRKY17* | 0.87 | *WRKY* |
| *BraA02g028540.3C* | *BrWRKY41* | -0.61 | *WRKY* |
| *BraA09g040790.3C* | *BrRAP2-13* | -0.66 | *ERF/AP2* |
| *BraA10g001670.3C* | *BrbHLH7* | -0.69 | *bHLH* |
| *BraA07g001000.3C* | *BrOZF1* | -0.77 | *OZF* |
| *BraA09g011350.3C* | *BrHBI1* | -0.88 | *bHLH* |
| *BraA07g009390.3C* | *BrbHLH77* | -0.9 | *bHLH* |
| *BraA08g003010.3C* | *BrSCL3* | -0.96 | *GRAS* |
