# Supplementary figures and images for: Effects of different light intensity on leaf color changes in a Chinese cabbage yellow cotyledon mutant
Source: Front Plant Sci. 2024 Apr 16;15:1371451. doi: 10.3389/fpls.2024.1371451 (PMC11058996; doi:10.3389/fpls.2024.1371451)

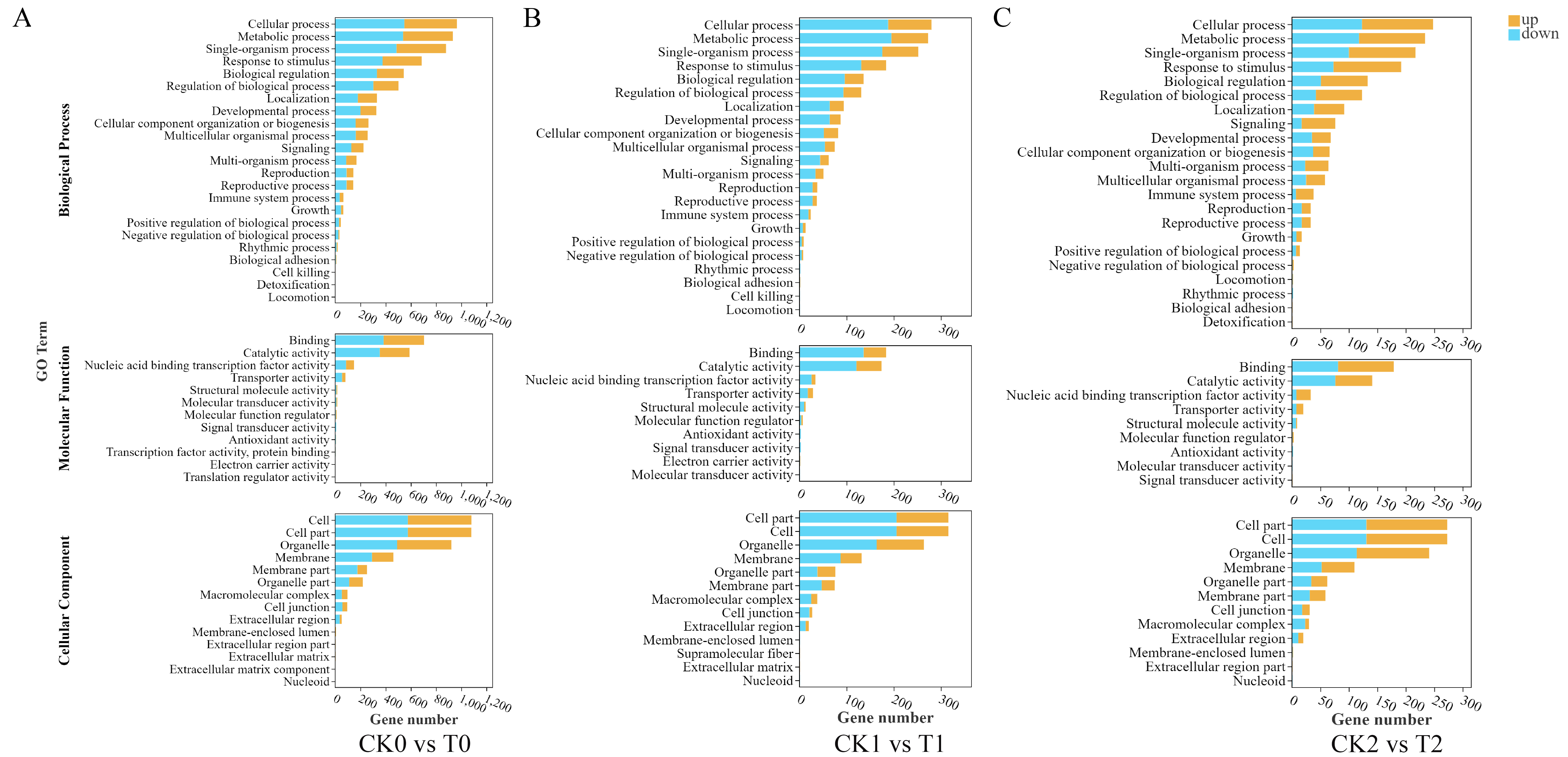

Supplement: Supplementary Figure 1 — Enrichment analysis of the identified DEGs based on GO terms. CK0, T0, CK1, T1, CK2, and T2 represent the six cDNA libraries from 19GC-2 and 19YC-2. [file Image_1.jpeg]

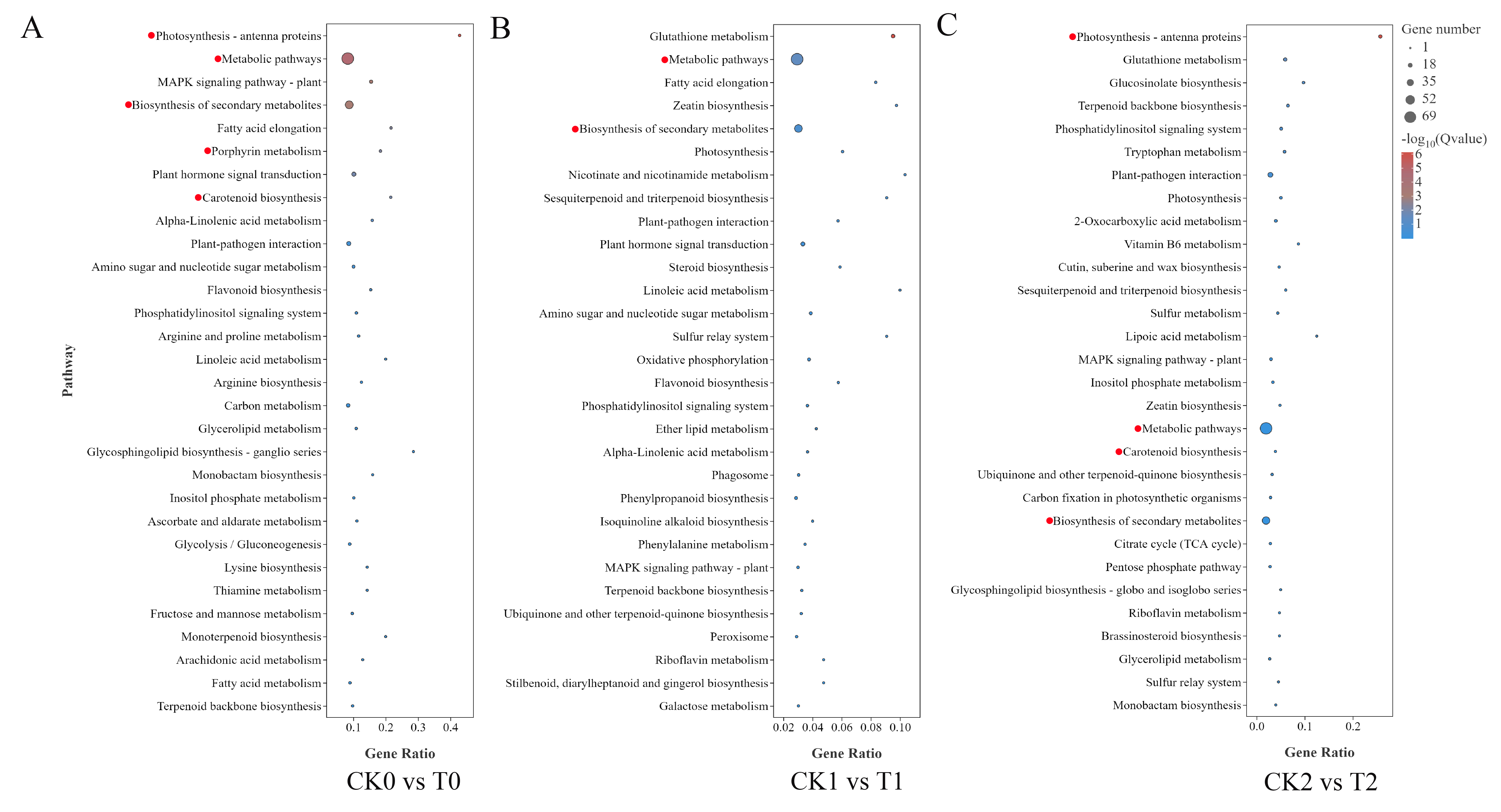

Supplement: Supplementary Figure 2 — Top 30 enriched KEGG pathways of the identified DEGs. CK0, T0, CK1, T1, CK2, and T2 represent the six cDNA libraries from 19GC-2 and 19YC-2. Red circles indicate the critical pathways. [file Image_2.jpeg]

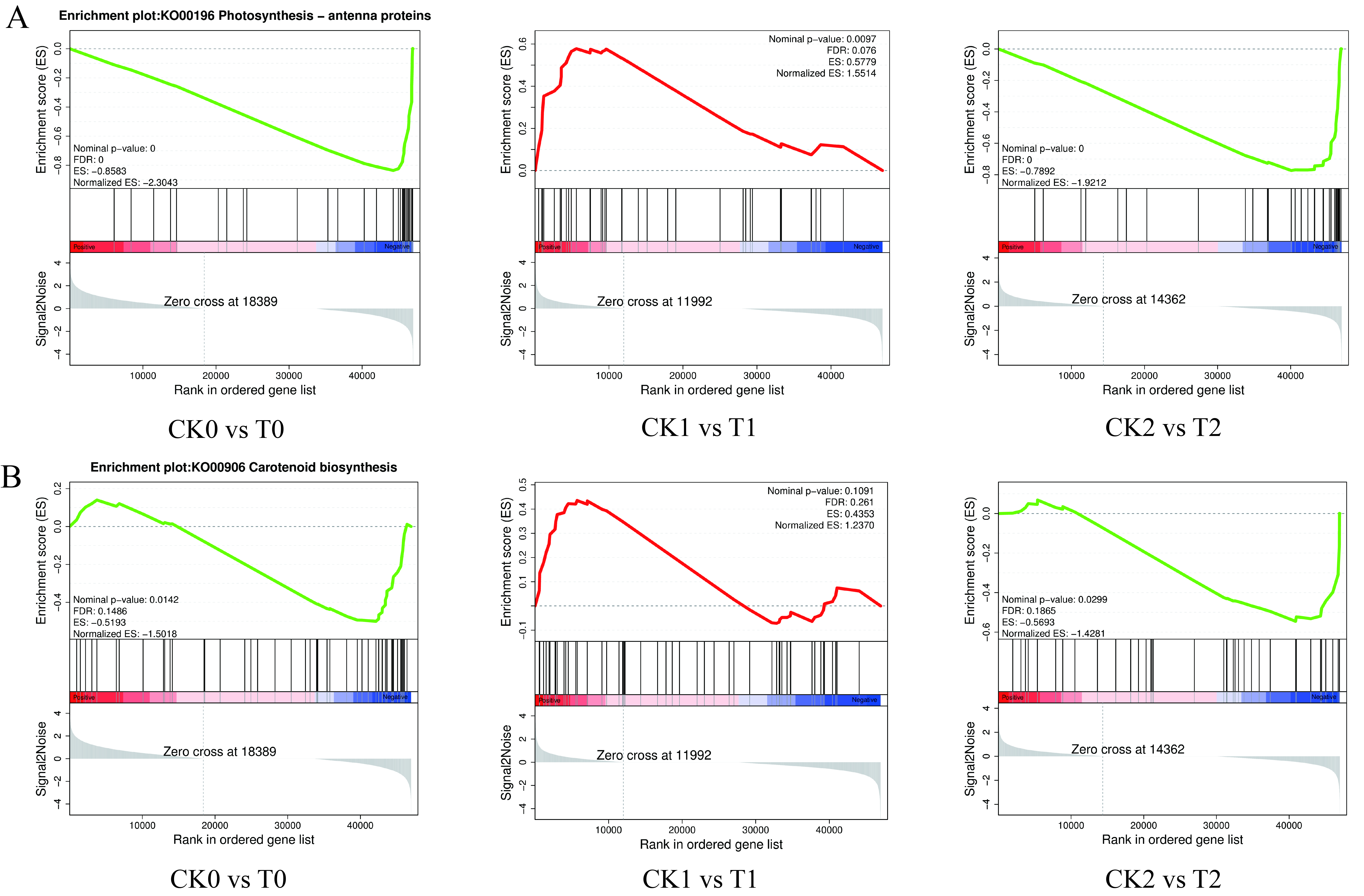

Supplement: Supplementary Figure 3 — GSEA analysis of the pathways of photosynthesis antenna proteins (A) and carotenoid biosynthesis (B). CK0, T0, CK1, T1, CK2, and T2 represent the six cDNA libraries from 19GC-2 and 19YC-2. [file Image_3.jpeg]
